# Hippocampal Changes Elicited by Metabolic and Inflammatory Stressors following Prenatal Maternal Infection

**DOI:** 10.3390/genes14010077

**Published:** 2022-12-26

**Authors:** Sandra L. Rodriguez-Zas, Bruce R. Southey, Haley E. Rymut, Laurie A. Rund, Rodney W. Johnson

**Affiliations:** 1 Department of Animal Sciences, University of Illinois at Urbana-Champaign, Urbana, IL 61801, USA; 2 Neuroscience Program, University of Illinois at Urbana-Champaign, Urbana, IL 61801, USA; 3 Division of Nutritional Sciences, University of Illinois at Urbana-Champaign, Urbana, IL 61801, USA; 4 Department of Statistics, University of Illinois at Urbana-Champaign, Urbana, IL 61801, USA

**Keywords:** cAMP, postnatal stress, networks

## Abstract

The hippocampus participates in spatial navigation and behavioral processes, displays molecular plasticity in response to environmental challenges, and can play a role in neuropsychiatric diseases. The combined effects of inflammatory prenatal and postnatal challenges can disrupt the hippocampal gene networks and regulatory mechanisms. Using a proven pig model of viral maternal immune activation (MIA) matched to controls and an RNA-sequencing approach, the hippocampal transcriptome was profiled on two-month-old female and male offspring assigned to fasting, mimetic viral, or saline treatments. More than 2600 genes presented single or combined effects (FDR-adjusted *p*-value < 0.05) of MIA, postnatal stress, or sex. Biological processes and pathways encompassing messenger cyclic adenosine 3′,5′-monophosphate (cAMP) signaling were enriched with genes including gastric inhibitory polypeptide receptor (GIPR) predominantly over-expressed in the MIA-exposed fasting males relative to groups that differed in sex, prenatal or postnatal challenge. While this pattern was amplified in fasting offspring, the postnatal inflammatory challenge appeared to cancel out the effects of the prenatal challenge. The transcription factors C-terminal binding protein 2 (CTBP2), RE1 silencing transcription factor (REST), signal transducer and activator of transcription 1 (STAT1), and SUZ12 polycomb repressive complex 2 subunit were over-represented among the genes impacted by the prenatal and postnatal factors studied. Our results indicate that one environmental challenge can influence the effect of another challenge on the hippocampal transcriptome. These findings can assist in the identification of molecular targets to ameliorate the effects of pre-and post-natal stressors on hippocampal-associated physiology and behavior.

## 1. Introduction

Infectious diseases and other environmental stressors may elicit inflammatory signals and molecular changes in the peripheral and central nervous systems. The female’s inflammatory response to challenges during gestation, known as maternal immune activation (MIA), may impact the molecular pathways of the developing offspring’s central nervous system [1] and can have long-lasting effects on the postnatal physiology and behavior [2,3]. Among the central nervous system structures, the hippocampus participates in processes associated with social and goal-oriented behavior, spatial memory, flexible cognition, and can play a role in neuropsychiatric diseases [4].

A study of MIA induced by the porcine reproductive and respiratory syndrome virus (PRRSV) during the final third of gestation reported elevated expression of inflammatory cytokine genes and low expression of major histocompatibility complex II genes in the fetal hippocampi five weeks post-infection [5]. Maternal immune activation continued to alter gene expression, and alternative splicing in the hippocampus and the amygdala interacted with stressors at three weeks of age [6,7]. The interaction of weaning and MIA elicited the under-expression of genes in the terpenoid backbone biosynthesis pathway in the hippocampus [8] and the neuroactive ligand-receptor pathway in the amygdala [9]. The effect of MIA on metabolic pathways was reported in a metabolomics study of MIA-exposed weaned pigs, including increased hepatic levels of cholesterol and glucogenic amino acid histidine [10]. While the stress elicited by weaning is a composite of environmental, physical, and social challenges, a characterization of the combined effects of MIA and specific challenges later in life is needed.

The present study aims to further the understanding of the combined effect of MIA and postnatal metabolic and inflammatory stressors on the hippocampus within and across sexes. The hippocampal transcriptome profiles of two-month-old female and male pigs exposed to MIA during gestation were benchmarked against controls using an RNA-sequencing approach. The effects of MIA were explored in three postnatal scenarios, fasting metabolic challenge, viral mimetic inflammatory challenge, or saline treatments. Network inference, gene pathway, and regulatory analyses further the understanding of the effects of postnatal stressors and MIA.

## 2. Materials and Methods

### 2.1. Animal Experiments

The animal studies were sanctioned by the Institutional Animal Care and Use Committee at the University of Illinois, and previously described protocols were used in this study [2,5,11]. Twenty healthy gilts from a PIC Camborough 22 genetic line (PIC, Hendersonville, TN, USA) were inseminated with semen from PIC 359 boars (PIC, Hendersonville, TN, USA). The gilts were transported from the University of Illinois swine herd to individual chambers in a biosafety facility with rooms maintained at 22 °C and exposed to a 12 h light cycle with lights on at 7:00 a.m. The gilts received a diet that satisfied the nutritional requirements at each trial stage and had ad libitum access to water, and half of them were challenged with live PRRSV (intranasal inoculation of strain P129-BV, School of Veterinary Medicine at Purdue University, West Lafayette, IN, USA). The PRRSV challenge (5 mL of 1 × 10^5^ median tissue culture infection dose) was diluted in sterile Dulbecco’s modified Eagle medium (5 mL total volume), and the remaining half of the gilts were inoculated with the sterile medium and served as controls. The virally challenged gilts developed a fever and lost appetite for approximately 12 days after inoculation reports, and the feed available to the controls was limited to the feed consumed by the MIA group the previous day [2,9].

Farrowing was induced to coincide with the expected gestation length of 113 days, and the offspring received the standard inoculations to manage iron levels and respiratory diseases [8,9]. The pigs remained with the mother in the individual crates until weaning at 21 days of age. The pigs were randomly assigned to rooms that housed four to five pigs each, received a nutritionally complete diet, and had ad libitum access to water [3,11]. At 59 days of age, 72 pigs of both sexes were randomly assigned to one of three postnatal challenge groups, (i) 24 h fasting; (ii) the following day, another third of the pigs received at approximately 7:00 AM an intraperitoneal injection of Poly(I:C) in a dose of 1.0 mg/kg of body weight (Sigma, St. Louis, MO, USA) following published protocols [2]; and (iii) the remaining third of the pigs were intraperitoneally injected with sterile phosphate-buffered saline in a volume comparable to the Poly(I:C) injection to ensure blind treatment assignment and were ascribed to the no stress, baseline saline-treated group. The three pig treatment groups (*n* = 24/group) represented metabolic stress, inflammatory stress, and baseline, respectively.

### 2.2. RNA Sequencing, Mapping, and Analysis

At approximately 100 days post-inoculation, the 60-day-old pigs were anesthetized intramuscularly and euthanized following published protocols [1,5,8]. The hippocampi from the 72 pigs were dissected, flash-frozen on dry ice, and stored at −80 °C, and the RNA was extracted using the kit EZNA (Omega Biotek, Norcross, GA, USA). The mRNA preparation kit TruSeq Stranded (Illumina Inc, San Diego, CA, USA) was used to develop the RNAseq libraries that were quantified using qPCR. Pair-end reads were sequenced on two lanes using a NovaSeq 6000 using the NovaSeq S4 kit. The demultiplexed FASTQ files, including the read sequences and associated quality scores, were obtained using the bcl2fastq v2.20 routine (Illumina Inc, San Diego, CA, USA).

Read quality profiling using FASTQC [12] indicated a minimum Phred quality score of 35 across the read positions, and sequence trimming was unnecessary. Paired-end sequences were mapped to the Sus scrofa genome version 11.1, and per-gene read abundance per gene was obtained using kallisto version 43.0 with default specifications [13]. The trimmed-mean normalized values of the genes detected with more than five transcripts per million and group were analyzed using the edgeR version 3.14 software [14]. The gene expression levels were tested for the main effects and interactions of MIA (MIA and control groups), postnatal stress at 60 days of age (a) fasting, (b) Poly(I:C) inflammatory stress, and (c) baseline saline groups), and sex (males and females). While the differential expression test for the effects with two levels (i.e., MIA or sex) encompassed one contrast and 1 degree of freedom, the test for postnatal stress encompassed two contrasts, Poly(I:C) versus saline baseline and fasting versus baseline. Therefore, the interactions encompassing postnatal stress have two estimates, each corresponding to a stressor relative to baseline. The effects that surpassed the False Discovery Rate-adjusted *p*-value < 0.05 [15] were considered statistically significant. In pairwise comparisons between groups, a positive (or negative) log_2_(fold change) indicates over-expression (or under-expression) in the first treatment group relative to the second group. The expression levels of the genes studied are available in the Gene Expression Omnibus database (GEO), number GSE220875.

### 2.3. Gene Ontology, Network Inference, and Transcription Factor Analysis

The enriched Gene Ontology biological processes among genes presenting MIA, stress, sex, or interaction effects (FDR-adjusted *p*-value < 0.05) were detected using the hypergeometric test in the over-representation analysis (ORA) routine of the WebGESTALT package [16]. Sus scrofa was selected as the reference organism for the enrichment analyses. The over-representation of Kyoto Encyclopedia of Genes and Genomes (KEGG) pathways among the genes presenting extreme over-expression or under-expression between treatment groups was analyzed using the Gene Set Enrichment Analysis (GSEA) routine of WebGESTALT. The ORA enrichment score represents the ratio between the observed and expected category representation on the gene list [16]. The GSEA normalized enrichment score (NES) represents the ratio between the observed score (maximum deviation of cumulative rank sum according to the gene signed fold change) relative to the expected value calculated from the average of 1000 permutated scores [16]. Categories supported by a minimum of 5 genes and FDR-adjusted *p*-value < 0.05 are discussed. For the main effects, the sign of the GSEA score follows the sign of the gene expression log_2_(fold change), where positive values indicate pathway enrichment among the genes over-expressed in the first group relative to the second group of samples. For the interactions, negative GSEA enrichment scores denote a synergistic mode of action between MIA and postnatal stress, MIA and males, or stress and males. In contrast, positive scores denote an antagonistic mode of action. Synergistic action is characterized by higher gene expression in the MIA stressed, MIA males, and stressed males relative to other groups. Conversely, antagonistic action is characterized by higher gene expression in groups exposed to either one prenatal MIA or postnatal stress challenge alone or females exposed to either the MIA or postnatal challenge.

The strength and direction of the co-expression between genes in pathways were investigated using the product-moment correlation coefficient between the log_2_(fold change) profiles and correlation *p*-values. The observed gene relationships were benchmarked against the STRING database v. 11.0 [17], which integrates molecular relationship evidence from experiments, sequences, and literature text mining using the human genome as a reference. The observed correlations reaching a *p*-value < 0.05 were compared to the STRING molecular relationships supported by text mining, co-expression, and experiment databases with a default confidence score cutoff >0.4 [17]. The cutoff threshold corresponds to medium confidence that prioritizes sensitivity, while a false positive rate of 1 is achieved with the *p*-value cutoff.

The network of nodes representing genes and edges representing significant and expected co-expression patterns were visualized using the STRING application in the Cytoscape v. 3.8.1 environment [18]. The color of the node reflects the sign of the fold change where red (green) denotes under- (over-) expression in the first relative to the second group of pigs compared (differing in MIA, stress, or sex), and yellow identifies less extreme differential expression between the groups.

The transcription factors and regulatory motifs shared by the genes that have significant MIA, stress, sex, or interaction effects (FDR-adjusted *p*-value < 0.0005) were identified using the iRegulon application and the human genome as a reference in Cytoscape [19]. The stringency on the gene lists ensured that the regulatory enrichment was supported by substantial changes in expression across MIA, stress, and sex groups. Transcription factors and motifs were deemed enriched at the default normalized enrichment score > 3 and area under the cumulative recovery curve (AUC) > 0.03. This indicator is computed for each motif at the initiation of the cumulative gene recovery plot (akin to a receiving operator curve) of the input gene detection along the genome. These computations correspond to FDR-adjusted *p*-value < 0.01 and approximately 400 top-ranked input genes [19].

## 3. Results

### 3.1. Sequence and Annotation Statistics

The minimum RNA integrity number across the 72 samples was 7.1, and the RNA sequencing produced an average of 170 million single-end reads (or 85.02 million paired-end reads) per sample. The coefficient of variation of the number of reads per sample was approximately 0.13, indicating that the sequencing efficiency was comparable among sexes, postnatal stress, and MIA groups. The average percentage of reads mapped per sample was 77%, and 16,269 genes were analyzed for the effects of MIA, postnatal stress (inflammatory, fasting, baseline no stress), sex, and associated interactions.

Overall, 2618 genes presented one or more significant (FDR-adjusted *p*-value < 0.05) main or interaction effect. More than fifty percent of the differentially expressed genes were detected for MIA either interacting with the postnatal stress (677 genes) and sex (249 genes) or independent of other factors (533 genes). Additionally, detected were the effects of postnatal stress interacting with sex (430 genes), the independent effects of the stress (421 genes), and sex (308 genes). Supplementary Table S1 lists the genes differentially expressed at FDR-adjusted *p*-value < 0.05 and |log2(fold change between treatment groups)| > 2 across the effects tested.

### 3.2. Effects of Maternal Immune Activation, Postnatal Stress, and Sex on the Gene Pathways in the Hippocampus

The complementary enrichment analyses identified functional categories enriched among the genes presenting MIA, postnatal stress, and sex effects in interacting and independent modes of action. Table 1 summarizes the biological processes enriched from the ORA analysis among genes presenting significant MIA (M), postnatal stress elicited by Poly(I:C) or fasting (PF), sex (S), or interaction (x) effects (FDR-adjusted *p*-value < 0.05) in the hippocampus. Complementing the ORA enrichment results, Table 2 lists the enriched KEGG pathways from the GSEA analysis of all genes that account for the signed logarithm fold change. Supplementary Table S2 includes a more comprehensive list of enriched (FDR-adjusted *p*-value < 0.1) biological processes among the differentially expressed genes. A negative GSEA enrichment score for the interaction MIA-by-Poly(I:C) and positive score for the Poly(I:C) effect indicate that the expression of most genes in the category was higher in individuals exposed to both relative to individuals exposed to either challenge. A comparable interpretation can be applied to the interactions sex-by-MIA or sex-by-stress, such that a negative (positive) enrichment score denotes that males (females) exposed to MIA or males (females) exposed to stress have higher gene expression relative to other groups.

Many enriched biological processes and pathways included cyclic adenosine 3′,5′-monophosphate (cAMP) signaling molecules or cAMP-responsive elements. The consistency between the ORA and GSEA analyses of genes presenting MIA and postnatal stress effects is evidenced in the enrichment of processes and pathways associated with regulating cAMP-mediated signaling or encompassing cAMP-responsive elements and cAMP-activated molecules. Additionally, ORA and GSEA shared processes and pathways associated with inflammation and immune challenge (e.g., chemokine signaling), hormone processing (e.g., thyroid hormone), and neural signaling (e.g., synaptic and postsynaptic signaling).

The results from the GSEA approach indicate that the enrichment of categories among genes presenting MIA-by-postnatal stress interaction is associated with genes presenting differential expression in response to the Poly(I:C) challenge (*p* effect) relative to fasting (F effect) in Table 2. Additionally, the predominance of a positive enrichment score sign for *p* indicates that most of the enrichment was associated with over-expression in Poly(I:C) relative to baseline saline-treated pigs. Conversely, the predominance of a negative enrichment score sign for the effect of sex indicates that the genes were over-expressed in females relative to males.

### 3.3. Effects of Maternal Immune Activation, Postnatal Stress, and Sex on Gene Expression in the Hippocampus

The functional analysis results allowed focus on the study of genes annotated to the enriched categories and impacted by MIA, postnatal stress, or sex. Table 3 summarizes the log_2_(fold change) of genes in inflammatory, neural, and hormonal functional categories (I, N, and H annotations, respectively) that were differentially expressed (FDR-adjusted *p*-value < 0.05 and |log_2_(fold change)| > 5) and cAMP-associated genes that were differentially expressed at FDR-adjusted *p*-value < 0.05 in at least one effect studied.

The genes in Table 3 include calbindin 1 (CALB1), cerebellin 3 precursor protein (CBLN3), γ-aminobutyric acid (GABRA6), G-protein coupled receptor 88 (GPR88), neuropeptide S receptor 1 (NPSR1), neurotensin (NTS), C-X-C motif chemokine ligand 2 (CXCL2), C-X-C motif chemokine ligand 8 CXCL8, selectin E (SELE), growth hormone 1 (GH1), prolactin (PRL), sine oculis-related homeobox 3 (Six3). Genes participating in cAMP signaling include adenylate cyclase type 5 (ADCY5), adenosine receptor A2a (ADORA2A), Calcium/calmodulin-dependent protein kinase type 4 (CAMK4), D (1A) dopamine receptor (DRD1), D (2) dopamine receptor (DRD2), gastric inhibitory polypeptide receptor (GIPR), 5-hydroxytryptamine receptor 1D (HTR1D), 5-hydroxytryptamine receptor 1F (HTR1F), transducer protein Htr6 (HTR6), pro-neuropeptide Y (NPY), cAMP and cAMP-inhibited cGMP 3’,5’-cyclic phosphodiesterase 10A (PDE10A), protein phosphatase 1 regulatory subunit 1B (PPP1R1B), and somatostatin receptor type 5 (SSTR5). Supplementary Table S1 includes a more comprehensive list of genes differentially expressed at FDR-adjusted *p*-value < 0.05 and |log_2_(fold change between treatment groups)| > 2 across the effects studied.

In general, the profiles of the genes associated with neural signaling (e.g., CALB1) presented positive expression profiles indicating that these genes were over-expressed in conditions with either the postnatal (Poly(I:C) or fasting) stress relative to saline and under-expressed in MIA relative to control. Inflammation-related genes, including CXCL2 and CXCL8, were over-expressed in postnatal Poly(I:C) stress relative to saline and in fasting males. Genes annotated to hormone-related functions (e.g., GH1) were over-expressed in MIA relative to control and in postnatal stress conditions relative to saline. Genes associated with cAMP-related activities (e.g., DRD1 and DRD2) were over-expressed in fasting MIA males relative to other groups.

### 3.4. cAMP Signaling Network Profiles Impacted by Maternal Immune Activation, Postnatal Stress, and Sex

**Figure 1:**
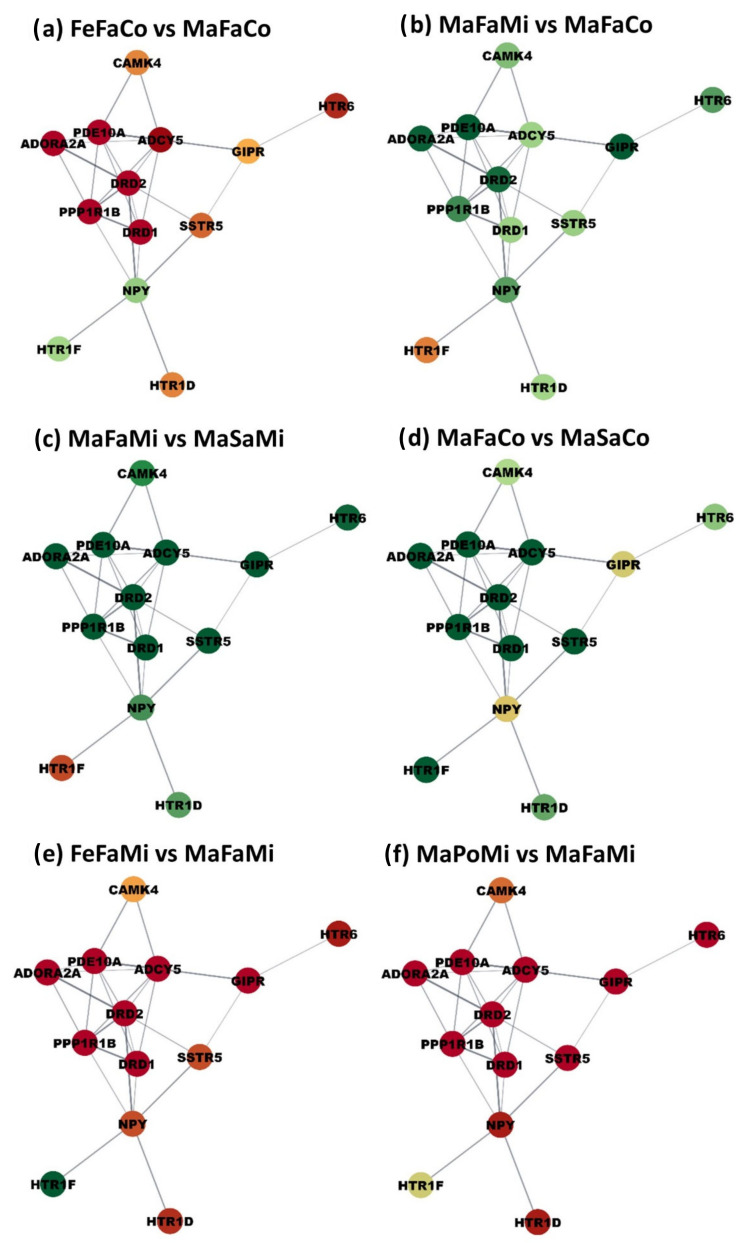
Gene networks for the cAMP signaling pathway showcasing prevalent, consistent, and extreme effects of maternal immune activation, postnatal stress, and sex across the genes. Nodes represent the genes; edges represent STRING database connections confirmed by the study; node color represents differential expression between groups. The first letters of the contrasts label denote the sex (Ma = male vs. Fe = female), followed by the postnatal stressor (Sa = saline vs. Po = Poly(I:C) vs. Fa = fasting), and the ending letters denote the maternal immune activation group (Co = non-MIA controls vs. Mi = MIA PRRSV-exposed animals). Colors range from red (under-expression in the first group vs. the second group) to yellow (neutral fold change value) to green (over-expressed in the first group vs. the second group).

**Figure 2:**
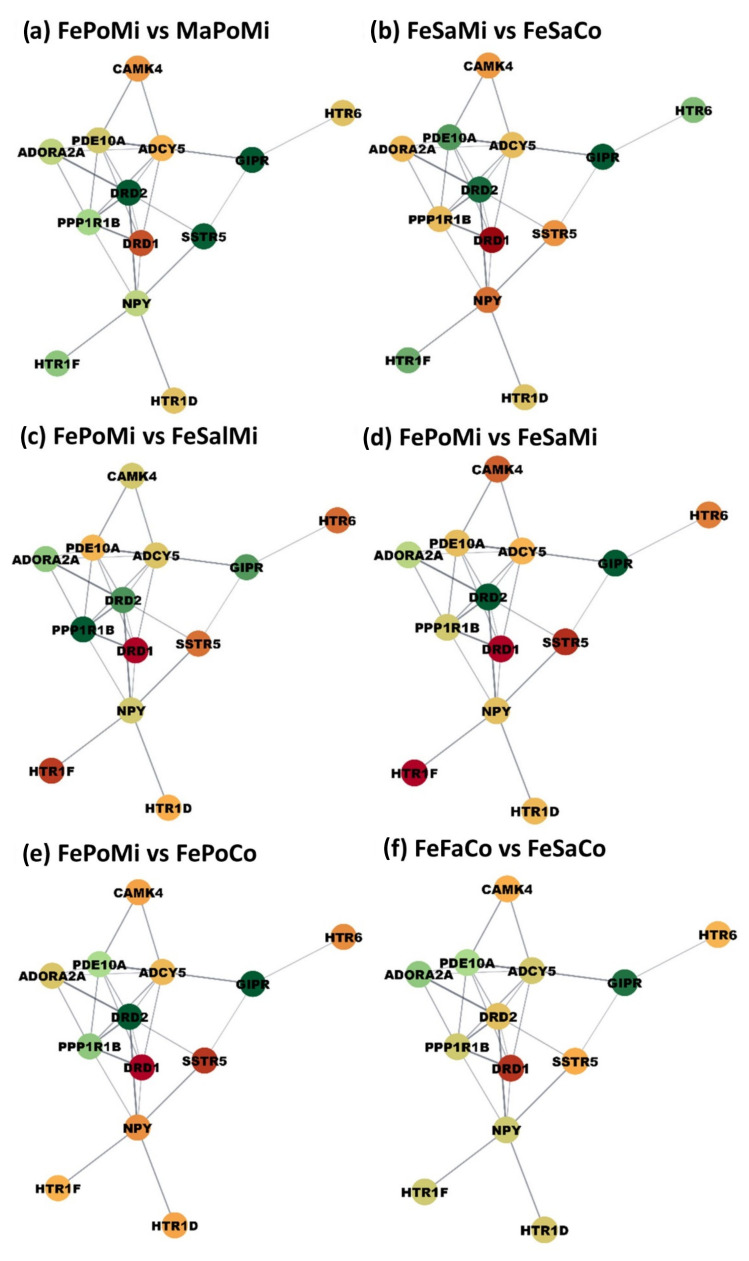
Gene networks for the cAMP signaling showcasing group contrasts presenting a wide range of gene expression changes across the network. Nodes represent the genes; edges represent STRING database connections confirmed by the study; node color represents differential expression between groups. The first letters of the contrasts label denote the sex (Ma = male vs. Fe = female), followed by the postnatal stressor (Sa = saline vs. Po = Poly(I:C) vs. Fa = fasting), and the ending letters denote the maternal immune activation group (Co = non-MIA controls vs. Mi = MIA PRRSV-exposed animals). Colors range from red (under-expression in the first group vs. the second group) to yellow (neutral fold change value) to green (over-expressed in the first group vs. the second group.

Results from the functional analysis indicated that MIA and postnatal stress impacted the expression of genes participating in cAMP signaling processes. The co-expression of genes annotated to the cAMP signaling process was investigated. All the expected gene relationships listed in the STRING database were observed except for GIPR with SSTR5 and HTR1F with NPY. Based on the overwhelming alignment between the observed and expected gene relationships, the networks depicted in Figure 1 and Figure 2 include the STRING database connections. Six contrasts between groups that showcase extreme changes in gene expression that have a shared profile and are prevalent across the cAMP signaling network are highlighted in Figure 1. Six contrasts between groups characterized by genes presenting a wide range of profiles between both extremes are highlighted in Figure 2. The network nodes represent the cAMP genes, and the edges represent significant co-expression patterns. Twelve networks depicting differences in gene expression between pairs of treatment groups that differ in one factor were visualized. Supplementary Table S3 presents the log_2_(fold change) values corresponding to the 24 possible contrasts between treatment groups.

The networks highlight sex interactions with MIA and postnatal stress previously identified in the enrichment analysis. For example, while there was overall over-expression of cAMP genes between fasting and non-stressed males from control gilts (Figure 1d), genes did not present a unified profile in females from control gilts (Figure 2f. Additionally, among control fasting offspring, all cAMP genes were over-expressed in males relative to females. Similar gene patterns were observed in offspring exposed to MIA, with gene over-expression in fasting males relative to Poly(I:C) males and in fasting females relative to saline-treated males.

Within the cAMP signaling network, the submodule encompassing genes DRD1, DRD2, and GIPR presented similar expression profiles across MIA, postnatal stress, and sex groups relative to other genes in the network. Among the three genes, DRD2 and GIPR present the most similar expression pattern (Supplementary Table S4). Additionally, presenting similar profiles across samples are the genes ADCY5, ADORA2A, DRD1, and SSTR2. Lastly, NPY follows the expression pattern of most genes in the network.

The profiles of the module genes DRD1, DRD2, and GIPR were characterized by under-expression in MIA-exposed Poly(I:C)-challenged females relative to control Poly(I:C)-challenged females, MIA-exposed fasting females, MIA-exposed saline-treated females, Mia-exposed Poly(I:C)-challenged males, and control Poly(I:C)-challenged males. Additionally, in control non-stressed females, DRD1, DRD2, and GIPR were over-expressed relative to MIA-exposed non-stressed females, control fasting females, and control non-stressed males.

The expression pattern of HTR1F tended to be opposite to most of the other genes in the cAMP signaling network. Additionally, HTR1F presented a more extreme differential expression between sample groups under some conditions, including over-expression in MIA-exposed fasting females relative to MIA-exposed fasting males, MI-exposed A Poly(I:C)-challenged females, and control fasting females. Conversely, HTR1F was under-expressed in control saline-treated males relative to MIA-exposed saline-treated males, control saline-treated females, and MIA-exposed fasting males. Lastly, HTR1F was over-expressed in MIA-exposed fasting males relative to saline-treated offspring.

### 3.5. Regulatory Motifs and Transcription Factors Associated with Genes Impacted by Maternal Immune Activation, Postnatal Stress, and Sex in the Hippocampus

Further insight into the interrelationship among the genes influenced (FDR-adjusted *p*-value < 0.005) by MIA, postnatal stress, and sex acting alone or interacting was gained by identifying shared transcription factors and regulatory motifs. Table 4 lists the number of differentially expressed gene targets corresponding to the top five transcription factors characterized by an NES > 3 and an AUC > 0.03. Supplementary Table S4 includes the list of transcription factors enriched (normalized enrichments scores NES > 3, AUC > 0.03) from the genes influenced (FDR-adjusted *p*-value < 0.005) by MIA, postnatal stress, and sex acting alone or interacting with each other. Supplementary Figure S1 depicts the relationship between the transcription factors and the differentially expressed target genes for each effect studied.

The study of transcription factor enrichment offered insights into the potential impact of MIA, postnatal stress, and sex on gene regulatory mechanisms that are not necessarily mediated by significant changes in the expression of the transcription factor genes. The most differentially expressed transcription factor genes included RE1 Silencing Transcription Factor (REST), which was over-expressed in MIA relative to control pigs (*p*-value < 0.05), and signal transducer and activator of transcription 1 (STAT1) that presented significant MIA-by-postnatal stress and sex effects (*p*-value < 0.05). Most transcription factors are enriched on genes impacted by one or two factors such as SWI/SNF related, matrix associated, actin dependent regulator of chromatin, subfamily a, member 4 (SMARCA4) that is enriched for the effects of MIA and MIA-by-sex. On the other hand, REST and SUZ12 polycomb repressive complex 2 subunit (SUZ12) regulate genes that were differentially expressed across multiple factors studied.

## 4. Discussion

The present study advances the understanding of the effects of prenatal virally elicited MIA interacting separately with the metabolic stress of fasting and the inflammatory challenge elicited by viral mimetic Poly(I:C) on two-month-old pigs. The majority of the differentially expressed genes (FDR-adjusted *p*-value < 0.05) were associated with the effect of MIA, alone or interacting with the postnatal stress or sex (1460 genes) and sex (249 genes). Overall, 1160 genes presented significant effects of postnatal stress and sex alone or through interactions. These overarching metrics speak to the prolonged effect of MIA and the importance of considering the potential synergistic or antagonistic effects with subsequent stressors. Additionally, important in studying prenatal and postnatal stressors is the consideration of sex-dependent effects.

### 4.1. Processes and Pathways Impacted by Maternal Immune Activation, Postnatal Stress, and Sex

The investigations into the biological processes and pathways indicated that genes participating in cAMP-related mechanisms were impacted by MIA, postnatal stress, and sex (Table 1 and Table 2). Among the 12 cAMP-related enriched categories was the biological process of positive regulation of cAMP-mediated signaling encompassing genes that were impacted by postnatal stress alone or interacting with MIA and sex. Considering that fasting was a postnatal stressor evaluated, the present finding aligns with reports that the cAMP response element-binding protein (CREB) is activated during intermittent fasting and participates in neurogenesis and energy metabolism processes [20]. Our findings about the impact of postnatal Poly(I:C) stress on cAMP-related processes (e.g., Staphylococcus aureus infection, tumor necrosis factor -TNF- signaling, regulation of endothelial cell apoptosis, and regulation of angiogenesis) relates to reports that disruption of CREB function in adult forebrain neurons is associated with establishment in the hippocampus of sub-inflammatory conditions [21]. The positive enrichment score of the pathways encompassing cAMP-related processes (e.g., TNF signaling) indicates that the genes in these categories were over-expressed in postnatal stress relative to saline conditions. The higher expression of genes associated with cAMP-related activities may support the lowering of proinflammatory conditions in the pig elicited by the Poly(I:C) challenge at two months of age. The previously observed profile is consistent with reports that deletion or lower levels of CREB in the hippocampus result in the recruitment of cell types and molecules responsible for neuroinflammation [21].

Consistent with the present enrichment results, a meta-analysis of studies on MIA-related schizophrenia spectrum disorder in humans identified enrichment of cAMP-mediate signaling, calcium signaling, T cell receptor signaling, nicotine addiction, and morphine addiction [22]. The enrichment of the biological processes of cellular response to interferon-γ (IFN-γ) and interleukin 1 among the genes affected by postnatal stress is consistent with higher expression levels of IFN-γ, and its receptor in the hippocampus of rats receiving an intermittent fasting regimen relative to rats fed ad libitum [23]. The findings from the present study are aligned with the idea that dietary restrictions can elicit a modest cellular stress response and neuroprotection through the release of neurotrophic factors. The hippocampal neuronal system adapts to intermittent fasting by improving the GABAergic tone, resulting in lower anxiety-like behaviors and improved hippocampus-dependent memory [23].

The enrichment of chemokine signaling among genes impacted by postnatal stressors is also consistent with reports that IFN-γ can protect neural cells against the detrimental effects of viral infections and associated inflammatory responses [23]. In addition, IFN-γ protected the hippocampal neurons against excitotoxicity involving JAK-STAT mechanisms. Neuronal treatment with IFN-γ resulted in the prompt recovery of the intracellular calcium levels following a glutamate challenge, suggesting that IFN-γ enhances calcium extrusion and buffering [23]. These observations were consistent with our findings of enrichment of the positive regulation of the JAK-STAT cascade and regulation of the release of sequestered calcium among the genes impacted by the postnatal stressors evaluated in the present study. 

A synergistic mode of action between the challenges was identified for the enriched categories among the genes with significant MIA-by-Poly(I:C) stress interaction effect. The negative GSEA of categories such as TNF and interleukin 17 signaling scores indicate elevated expression levels in the groups exposed to either MIA or postnatal Poly(I:C). This result suggests that MIA makes the hippocampus more tolerant to a second stress. Enrichment of related immune response pathways (e.g., antigen processing, Staphylococcus, and Herpes infection) was also observed in the study of three-week-old pigs exposed to MIA and the stress of weaning [8].

The positive GSEA score of immuno-related categories (e.g., Staphylococcus aureus infection and natural killer cell-mediated cytotoxicity) for the Poly(I:C)-by-sex interaction indicates that MIA-exposed males had higher expression levels of immuno-related genes relative to control males or females. Similar patterns were observed in MIA Poly(I:C)-exposed mice challenged with lipopolysaccharide after birth that resulted in higher mRNA levels of neuroinflammatory genes in the prefrontal cortex, amygdala, hippocampus, and thalamus of male relative to female mice and saline-treated males [24].

The positive enrichment scores of the categories detected in the MIA-by-fasting interaction indicate a synergistic effect, whereas groups exposed to one challenge (either MIA or fasting) had lower expression levels than groups exposed to both challenges or no challenge. This finding suggests that MIA primes the hippocampus to be more sensitized to the effects of fasting at two months of age. The previous result is consistent with the enrichment profiles corresponding to the interaction between MIA and weaning stress in pigs at three weeks of age [8]. While weaning encompasses multiple stressors, such as a change in feeding, housing, and socialization that can also trigger an inflammatory response, the results of the present study suggest that the previously reported interaction between MIA and weaning may be driven by changes in feed and food intake.

A noteworthy finding was that the pathways of taste transduction and nicotine addiction were enriched among the genes presenting MIA-by-fasting effect that was not enriched for the MIA-by-Poly(I:C) and MIA effects. The enrichment of the taste transduction pathway among genes presenting MIA-by-fasting interaction effects is consistent with reports that a link between nutritional status and the expression of taste receptors genes in the mouse hypothalamus [25]. Additionally, sweet taste transduction in the brainstem of mice was associated with the hypothalamic neuronal network regulating hunger-induced taste modification [26]. In overnight fasting mice, the agouti-related peptide-expressing neurons in the lateral hypothalamus can modify the aversive tasting signals. The alternation of taste preference seems to enhance calorie-rich diet consumption through networks that regulate hunger-induced taste modification in mice. Similarly, taste perception change was observed in patients diagnosed with type II diabetes or obesity relative to lean individuals [27].

The enrichment of the nicotine addiction pathway in the present study may be related to the enrichment of the morphine addiction pathway in the study of younger pigs experiencing the stress of weaning [8]. On the other hand, the enrichment of the terpenoid backbone biosynthesis and morphine addiction in the three-week-old MIA-exposed and weaned pigs did not reach statistical significance in the present study. Positive enrichment of these addiction pathways suggests the differential expression of shared genes underlying addiction are differentially expressed across ages and postnatal stressors in response to MIA.

The most enriched categories associated with sex effects had negative scores and had immune-related functions such as Leishmaniasis, Staphylococcus aureus infection, B cell receptor signaling, and viral myocarditis. The enrichment sign of these categories was also positive for significant Poly(I:C)-by-sex interaction effects. The previous combination of signs indicates that the genes were over-expressed in saline-treated females relative to Poly(I:C)-treated females and males irrespective of treatment. Over-expression of genes annotated to immune-related pathways in the hippocampus of female relative to male pigs were also observed in three-week-old pigs [8]. Our findings point to the hippocampus of female pigs responding with more extreme increases than saline-treated female or male pigs in the levels of expression of genes associated with an immune response to the Poly(I:C) challenge at two months of age. Similarly, a study of sexual dimorphism in the immune system transcriptome concluded that the stronger immune response of females relative to males could be associated with activated innate immune pathways [28].

### 4.2. Genes Impacted by Maternal Immune Activation, Postnatal Stress, and Sex

Complementing the study of enriched functional categories, an evaluation of numerous genes presenting extreme fold changes in association with MIA, postnatal stress, sex, and interactions was undertaken (Table 3). A cluster of genes presenting similar expression profiles were annotated to neurological pathways, including, Calbindin 1 (CALB1), Cerebellin 3 (CBLN3), and γ-aminobutyric acid type A receptor subunit alpha6 (GABRA6). These genes were overexpressed in MIA pigs exposed to postnatal stress compared to non-stressed conditions.

CALB1 is a marker for neuronal plasticity, and consistent with our findings, upregulation of CALB1 was reported in the cortical interneurons of individuals diagnosed with MIA-related schizophrenia spectrum disorder [29]. The significant interaction between MIA and either Poly(I:C) or fasting on CALB1 was characterized by over-expression in either prenatal (MIA) or postnatal challenge compared to no MIA or stressor. The strong over-expression of CALB1 in fasted or MIA-exposed pigs relative to non-fasting controls agrees with the over-expression of CALB1 in the striatum of four-month-old mice under dietary restriction relative to mice fed ad libitum [30]. Likewise, 8-week-old mice challenged with lipopolysaccharide presented higher expression levels of CALB1 than control mice in the dentate gyrus [31]. The previous associations are related to the role of CALB1 in the modulation of synaptic transmission, maintenance of calcium homeostasis, reduction of neuronal death, and elimination of neurotoxic products induced by proinflammatory cytokines [29].

CBLN3 is involved in the maintenance of synapse structure, and the pattern detected in the present study is aligned with the proposition that higher expression levels of CBLN3 offer protection against neuroinflammation [32]. The expression pattern of CBLN3 was similar to CALB1, including significant MIA-by-postnatal stress interaction, including a more extreme over-expression in either MIA or fasting followed by Poly(I:C). The over-expression of CBLN3 in fasting conditions is consistent with reports that CBLN3 was over-expressed in the hypothalamus of mice that had disrupted glucose metabolic homeostasis [33].

GABRA6 is annotated to multiple enriched pathways, including taste transduction and nicotine addiction, and has been linked to the susceptibility and pathophysiology of the MIA-associated schizophrenia spectrum disorder [34,35]. GABRA6, in addition to CALB1 and CBLN3, presented significant MIA-by-postnatal stress interactions characterized by over-expression in either the prenatal or postnatal stressor with fasting eliciting a more extreme differential expression than Poly(I:C). The effect of fasting on the hippocampus expression of GABRA6 may be associated with findings that a mutation in the human GABRA6 gene could be responsible for abnormalities of the hypothalamus homeostasis resulting in dysfunction in the glucose, insulin, and lipid metabolisms [36].

A group of genes annotated to immune pathways profile, including SELE, CXCL2, and CXCL8, presented a similar profile characterized by significant over-expression in the hippocampus of Poly(I:C) relative to the saline-treated group. Similar to our findings, an mRNA expression study of a model of proinflammatory mediators in the hippocampus of four-month-old rats reported that acute inflammatory response included over-expression of SELE [37]. The Poly(I:C)-dependent profile is consistent with the annotation of CXCL2 and CXCL8 to the TNF signaling pathway that participates in inflammation, immunity, and apoptosis processes. Our findings are also consistent with the role of endothelial-derived adhesion molecules (e.g., SELE) in promoting granulocyte recruitment and counteracting inflammation in the cerebral vasculature [38].

The pattern of genes annotated to the hormonal and neuroactive ligand-receptor interaction pathways, including GH1 and PRL, was characterized by synergistic over-expression in hippocampus exposed to both MIA and either one postnatal stressor studied. The pattern of the interaction between MIA and postnatal stress was consistent with the under-expression of GH1 in MIA-exposed pigs that were stressed (weaned) relative to non-stressed in the amygdala of 3-week-old pigs, while the opposite pattern was detected in pigs not exposed to MIA [9]. Additionally, consistent with the postnatal stress-by-sex results in the present study of hippocampus patterns in two-month-old pigs, GHI was under-expressed in the amygdala of 3-week-old weaned males relative to non-stressed pigs, whereas the pattern was opposite in females.

A similar profile to GH1 and PRL was observed in ATP synthase peripheral stalk subunit D (ATP5PD), a gene annotated to the neurodegeneration pathway. Consistent with the patterns in our study, ATP5PD is up-regulated in chronic inflammatory diseases [39]. The synergistic effect of MIA and postnatal stressors in the hippocampus of two-month-old pigs may be associated with the role of ATP5PD in the mitochondrial respiratory chain [40] and cumulative energy requirement to respond to MIA relative to postnatal stressors.

### 4.3. cAMP Signaling Network Profiles Impacted by Maternal Immune Activation, Secondary Stress, and Sex

The present study recovered the interconnections between cAMP signaling molecules listed in the STRING repository. The associations between DRD2, GIPR, DRD1, and SSTR5 in STRING and detected in this study are consistent with the participation of these molecules in G protein-coupled receptor signaling that plays a role in learning and memory and pain sensation [41]. The previous patterns agree with reports that the dopamine system in the hippocampus is overactive in patients diagnosed with MIA-related schizophrenia spectrum disorder [42]. This finding has led to the hypothesis that the responsive state of the dopamine system in the hippocampus plays an important role in behaviors characteristic of psychosis and MIA-associated schizophrenia spectrum disorder. The impact of MIA on cAMP signaling processes in the offspring hippocampus detected in the present study, and MIA-related behavioral disorders are consistent with changes in the behavior of MIA-exposed, 60-day-old pigs [2]. Behavior disruptions including changes in lateral and sternal laying, lethargy, touching, and walking were observed MIA-exposed pigs also challenged with Poly(I:C) and the effects were more marked in females [2].

The profiles of DRD2, GIPR, SSTR5, and DRD1 included over-expression in MIA-exposed fasting males relative to Poly(I:C)- and saline-treated males from control mothers (Figure 1, Supplementary Table S3). Additionally, the previous gene cluster was over-expressed in males from control mothers fasting relative to females and saline-treated males. These profiles are consistent with reports of over-expression of DRD1 in the hippocampus and DRD2 in the prefrontal cortex of juvenile mice exposed to Poly(I:C)-induced MIA [43,44]. Similarly, mice exposed to the inflammatory agent complete Freund’s adjuvant had higher GIPR expression in the anterior cingulate cortex, inhibiting neuroinflammation processes [45]. In alignment with the observed patterns, SSTR5 modulates the response to the stress of the hypothalamus-pituitary-adrenal axis and participates in inflammation response and hormone secretion [46].

Within the cAMP network module, including DRD2, GIPR, DRD1, and SSTR5, the profiles of DRD2 and GIPR were more similar to each other, and likewise, the profiles of DRD1 and SSTR5 were more similar to each other. The over-expression of GIPR in the hippocampus of MIA relative to control groups is consistent with reports that GIPR was up-regulated in the hippocampus of aging mice that presented other neuroinflammatory markers. This finding was related to studies indicating that GIPR knockout mice presented impaired learning and memory [47]. 

The expression profiles of DRD2 and GIPR were consistent across sexes and challenges, whereas the profiles of DRD1 and SSTR5 were reversed from DRD2 and GIPR in females under specific prenatal and postnatal challenges (Figure 2). The sex-dependent profile of SSTR5 is consistent with reports of sexually dimorphic changes in the expression of this somatostatin receptor in the brain of mice [48]. Likewise, the genomic of DRD1 presented sexual dimorphism in a study of MIA-related schizophrenia spectrum disorder [49], and rodent DRD1 were regulated in opposite directions in the two sexes after chronic stress [50].

The genes ADORA2A, PPP1R1B, DRD1, and DRD2 had a significant fasting-by-sex interaction effect and a less significant Poly(I:C)-by-sex interaction effect characterized by over-expression in challenged males or saline-treated females relative to other groups. This result is consistent with the downregulation of ADORA2A and DRD2 in the hippocampus of female mice exposed to a sub-chronic peripheral lipopolysaccharide challenge [51]. Additionally, consistent with the patterns observed in the present study, PPP1R1B was over-expressed in the hippocampus of unchallenged female relative to male mice [52].

The results from our network study indicate that MIA, postnatal stressors, and sex have the potential to exert a substantial effect on the expression of most genes in cAMP signaling processes. In general, the conditions of MIA, fasting, and males tended to enable a more extreme and overarching effect of the remaining factor on the genes in the cAMP signaling network. Notable was the consistent over-expression in MIA relative to control fasting males, over-expression between fasting and saline-treated males irrespective of prenatal exposure, the over-expression in fasting males relative to females irrespective of prenatal exposure, and the over-expression of fasting relative to Poly(I:C) MIA-exposed males. The cAMP networks depicting gene expression changes due to MIA and stressors in females tended to include more moderate profiles than in males. These findings suggest that prenatal or postnatal challenge conditions in females are more likely to have moderate effects or temper the effect of the remaining stressor.

### 4.4. Regulatory Motifs and Transcription Factors Impacted by Maternal Immune Activation, Postnatal Stress, and Sex

The study of transcription factors and motifs shared among differentially expressed genes (FDR-adjusted *p*-value < 0.005) in response to MIA, postnatal stress, and sex offered insights into regulatory sequences influencing hippocampal molecular processes. The visualization of the relationship between transcription factors through the common target genes suggests that most genes impacted by MIA and postnatal stress involved multiple enriched regulatory elements (Supplementary Figure S1). Distinct transcription factors targeted genes differentially expressed in response to sex, whereas multiple transcription factors targeted the same genes differentially expressed in response to MIA and postnatal stress. This finding suggests possible redundancy in the regulatory mechanisms of genes impacted by prenatal and postnatal challenges that could result in a more robust response to the studied challenges.

Many enriched transcription factors (e.g., REST, CTBP2, and SUZ12) have differentially expressed target genes associated with neurodevelopmental and neuroinflammation mechanisms. The detection of REST and SUZ12 is consistent with studies demonstrating the role of these factors as a master repressor of neuronal gene expression in mouse hippocampus during the neurodevelopment phase [53,54]. Likewise, nuclear factor interleukin-3 (NFIL3) has been associated with innate and adaptive immunity processes (i.e., natural killer cells, B cells, and macrophages) and immune-mediated diseases [55]. SMARCA4 has been associated with MIA-related autism spectrum disorder [56], and the nuclear factor kappa B subunit 1 (NFKB1) enrichment is aligned with the low-level chronic inflammation and enhanced response to an inflammatory stimulus in a knockout mouse model [57].

The present results indicate that some of the effects of maternal inflammatory signals during development and inflammatory and metabolic stressors on the hippocampus of 60-day-old pigs are distinct from transcriptome changes in response to prenatal and weaning stress in 21-day-old offspring. Subsequent studies can offer information on the hippocampus disruptions in the offspring at puberty and maturity. Additionally, the present study demonstrated the prolonged effect of MIA alone and interacting with secondary stressors and sex in the hippocampal molecular networks. The hippocampus participates in the inhibition of the hypothalamus-pituitary-adrenal (HPA) axis in response to stress, and the effect on the axis is dependent on the stimulus type [58]. Follow-up studies are needed to explore the impact of the prenatal and postnatal challenges on the HPA axis.

In conclusion, the network and functional analysis results indicate that one environmental challenge can influence the effect of another challenge on the hippocampal transcriptome. For the MIA and postnatal metabolic and inflammatory challenges evaluated in the present study, molecular pathways encompassing cAMP signaling were impacted by the combination of challenges at times in the hippocampus. The findings from the complementary approaches used in this study further the development of therapeutic strategies to mitigate the effect of multiple stressors at different stages of development.

## Figures and Tables

**Table 1:** Enriched (False Discovery Rate-adjusted *p*-value < 0.05) Gene Ontology (GO) biological processes identified using the over-representation analysis (ORA) among genes presenting significant (FDR-adjusted *p*-value < 0.05) maternal immune activation (M), postnatal stress (PF), sex (S), or interaction (x) effects in the hippocampus.

				Enrichment Ratio ^3^			
GO:Identity ^1^	Biological Process Name ^2^	MxPF	PFxS	MxS	M	PF	S
GO:0043950	positive reg. cAMP-mediated sig. *	23.6	23.4	56.9		19.9	50.1
GO:0051281	positive reg. release of seq. Ca				21.5		33.4
GO:0006911	phagocytosis, engulfment		31.2				
GO:0002548	monocyte chemotaxis					15.1	21.5
GO:0045744	negative reg. GPCR sig. pathway *		20.8				
GO:0042119	neutrophil activation		18.7			15.9	
GO:0071347	cellular resp. interleukin-1		12.5			16.9	
GO:1903169	reg. Ca transmembrane transport						16.2
GO:1904064	positive reg. cation transport				16.1		
GO:0048247	lymphocyte chemotaxis	11.8	12.5		16.1		
GO:0060078	regulation of postsynaptic potential						15.4
GO:0070098	chemokine-mediated sig. pathway		9.2			12.5	14.7
GO:0051279	reg. release of seq. Ca		12.5			10.6	
GO:2000351	reg. endothelial cell apoptosis *					12.0	
GO:0050810	reg. steroid biosynthetic process					11.8	
GO:0051928	positive reg. Ca ion transport	10.7					
GO:0002224	Toll-like receptor sig. pathway					9.8	
GO:0061844	antimicrobial humoral immune resp.					9.6	
GO:1904894	positive reg. STAT cascade					9.2	
GO:0046427	positive reg. JAK-STAT cascade					9.2	
GO:0071346	cellular resp.interferon-γ					8.8	
GO:0071356	cellular resp. tumor necrosis factor					8.7	
GO:0071774	response to fibroblast growth factor					8.5	
GO:0051607	defense resp. virus					6.9	8.4
GO:0120032	reg. plasma membrane projection				8.2		
GO:0071222	cellular resp. lipopolysaccharide	8.0				8.1	7.7
GO:0007189	adenylate cyclase-activate GPCR sig.		7.9		7.0	6.7	
GO:0030595	leukocyte chemotaxis				7.3		
GO:0097529	myeloid leukocyte migration	6.9					
GO:0043547	positive reg. GTPase activity					5.6	
GO:0046879	hormone secretion	4.8					

^1^ GO processes sorted from highest to lowest maximum enrichment ratio (ratio between observed and expected enrichment) across effects. ^2^ the “*” symbol denotes processes encompassing cAMP-related molecular mechanisms. Abbreviations: reg. = regulation of, seq. = sequestered, sig = signaling, resp = response, resp = response to. ^3^ enrichment ratio from the over-representation analysis.

**Table 2:** Enriched (False Discovery Rate -adjusted *p*-value < 0.05) Kyoto Encyclopedia of Genes and Genomes (KEGG) pathways identified using the Gene Set Enrichment Analysis (GSEA) among genes presenting high expression changes associated with maternal immune activation (M), postnatal stress of Poly(I:C) (*p*) or fasting (F), sex (S), or interaction effects in the hippocampus.

KEGG					Enrichment Score		
Identity ^1^	Pathway Name ^2^	MxF ^3^	MxP	PxS	M	*p*	S
ssc04668	TNF signaling *		−2.5			2.6	−2.1
ssc04657	IL-17 signaling		−2.2			2.5	−1.9
ssc04933	AGE-RAGE signaling in diabetes		−1.8	−1.8		2.4	
ssc04064	NF-kappa B signaling		−2.1			2.4	−2.0
ssc04742	Taste transduction *	2.3					
ssc04620	Toll-like receptor signaling		−2.0			2.2	−2.3
ssc04630	JAK-STAT signaling		−2.1	−1.9		2.2	
ssc05142	Chagas disease *		−1.7			2.2	−1.7
ssc05332	Graft-versus-host disease			2.2	1.8		
ssc04664	Fc epsilon RI signaling			2.1			
ssc05140	Leishmaniasis		−1.8	2.0		2.0	−2.1
ssc05150	Staphylococcus aureus infection *			1.9		1.9	−2.1
ssc05033	Nicotine addiction *	2.1					
ssc04917	Prolactin signaling		−1.9	−1.8		2.1	
ssc03050	Proteasome					−2.1	
ssc04672	Intestinal immune network for IgA			1.9			−2.1
ssc04662	B cell receptor signaling			2.1			−1.8
ssc05416	Viral myocarditis			2.0			−2.0
ssc04622	RIG-I-like receptor signaling		−2.0			2.0	−1.9
ssc04623	Cytosolic DNA-sensing		−1.9			2.0	
ssc04658	Th1 and Th2 cell differentiation		−1.8			2.0	−1.8
ssc05160	Hepatitis C					2.0	
ssc04918	Thyroid hormone synthesis *			−1.9			
ssc04512	ECM-receptor interaction			−1.9		1.9	
ssc05162	Measles		−1.8			1.9	−1.8
ssc04659	Th17 cell differentiation					1.9	−1.9
ssc04115	p53 signaling *					1.9	
ssc04650	Natural killer cell cytotoxicity			1.8			
ssc04744	Phototransduction			−1.8			
ssc04726	Serotonergic synapse *		−1.7				

^1^ pathways are sorted from highest to lowest maximum absolute normalized enrichment score (maximum deviation between observed and expected enrichment) value across effects. ^2^ the symbol “*” denotes pathways encompassing cAMP-related molecular mechanisms. ^3^ positive (or negative) enrichment score indicates gene over-expression (or under-expression) in the first group (MIA, male, and Poly(I:C) or fasting) relative to the second group compared (control, females, baseline), respectively.

**Table 3:** Log_2_(fold change) of differentially expressed (FDR-adjusted *p*-value < 0.05) genes in at least one interaction (x) or main effect of maternal immune activation (M), postnatal stressor of fasting (F) or Poly(I:C) (*p*), or sex (S) that are annotated to inflammatory (I), neural (N), and hormonal (H) functional categories in the hippocampus.

Signaling Process									
and Gene Symbol	M ^1^	F	*p*	S	MxF	MxP	FxS	PxS	MxS
Neuronal									
CALB1	3.0				7.0	2.0	4.1		2.4
CBLN3					5.0	1.5			
GABRA6				2.0	8.6	2.1			
GPR88							5.3		
NPSR1	−3.0	3.1	3.7	3.4			−3.0	−4.4	−7.5
NTS				−2.8			5.3		−2.2
Inflammatory									
CXCL2			7.1			−3.1			
CXCL8			6.7				2.7		2.3
SELE			8.3						
Hormonal									
GH1	−4.3	5.6	2.5		−3.3	−4.7	−6.1	−6.4	−7.5
PRL	−5.1	5.7	3.0	7.1	−3.5	−4.7	−5.4	−6.1	−8.5
SIX3							5.9		
cAMP									
ADORA2A							3.8		
DRD1							4.4		
DRD2							4.2		
GIPR	−1.5				−1.7				
PPP1R1B							3.1		

^1^ M = log_2_(MIA vs. control groups); S = log_2_(males vs. females); *p* = log_2_(Poly(I:C) vs. baseline groups); F = log_2_(fasting vs. baseline groups); MxF = log_2_(MIA fasting versus single challenge groups); MxP = log_2_(MIA Poly(I:C) versus single challenge groups); FxS = log_2_(fasting male versus fasting or male groups); PxS = log_2_(Poly(I:C) male versus Poly(I:C) or male groups); MxS = log_2_(MIA male versus MIA or male groups). Positive (or negative) log_2_(fold change) indicates gene over-expression (or under-expression) in the first group (MIA, male, and Poly(I:C) or fasting) relative to the second group compared (control, females, baseline), respectively.

**Table 4:** The number of target genes corresponding to the top 5 enriched (normalized enrichment score NES > 3, and area under the curve AUC > 0.03) transcription factors or motifs per factor studied among the genes affected (FDR-adjusted *p*-value < 0.005) by the effects of maternal immune activation (MIA), postnatal stress (PF), sex (S), or and the interactions (x) in the hippocampus.

Transcription		Number of Target Genes				
Factor ^1^	MIAxPF	PFxSex	MIAxSex	MIA	PF	S
REST		79	15	55		37
CTBP2		64		35		
STAT1	11					43
SUZ12	38	32	32	22		29
NFKB1					29	
NFIL3				28		
IRF1						26
TCF12			3		23	
EP300					19	
CHD1					18	
ETS2, NR3C1		18				
NFIC	18					
STAT2	12				14	17
RXRA	15					
TEAD4		14				
SMARCA4			7	13		
TP53			6			

^1^ transcription factors are sorted from highest to lowest number of target genes for the effect with the highest number of target genes.

## Data Availability

The raw and normalized gene expression levels are available in the National Center for Biotechnology Information (NCBI) Gene Expression Omnibus (GEO) database, series identifier GSExxxx (data submitted, identifier to be added once assigned).

## Supplementary Materials

The following supporting information is available online at https://www.mdpi.com/article/10.3390/genes14010077/s1, Supplementary Table S1: Genes differentially expressed at FDR-adjusted *p*-value < 0.05 and |log_2_(fold change between pig groups)| LFC > 2 across the tested effects of maternal immune activation (MIA), postnatal stress, sex and interactions (x); Supplementary Table S2: Gene Ontology biological processes enriched at FDR-adjusted *p*-value < 0.1 among genes differentially expressed (FDR-adjusted *p*-value < 0.05) in response to the effects of maternal immune activation (MIA), postnatal stress, sex, and corresponding interactions (x) detected using the hypergeometric over-representation analysis (ORA) in the hippocampus; Supplementary Table S3: Log_2_(fold change) values used to reconstruct the cAMP networks for the 24 pair-wise contrasts between groups characterized by the MIA (MiA, Control), stressor (Fast, Poly, Saline), and sex (Male, Female)and the baseline group; Supplementary Table S4: List of transcription factors and associated motifs enriched (normalized enrichments scores NES > 3, area under the curve AUC > 0.03) for the genes presenting effects (FDR-adjusted *p*-value < 0.005) of maternal immune activation (MIA), postnatal stress, sex, and interactions (x); Supplementary Figure S1: Relationship between the transcription factors and the differentially expressed target genes for each main effect (prenatal and postnatal stress and sex), and interactions obtained using iRegulon.
